# iCTX-Type: A Sequence-Based Predictor for Identifying the Types of Conotoxins in Targeting Ion Channels

**DOI:** 10.1155/2014/286419

**Published:** 2014-06-01

**Authors:** Hui Ding, En-Ze Deng, Lu-Feng Yuan, Li Liu, Hao Lin, Wei Chen, Kuo-Chen Chou

**Affiliations:** ^1^Key Laboratory for Neuro-Information of Ministry of Education, Center of Bioinformatics, School of Life Science and Technology, University of Electronic Science and Technology of China, Chengdu 610054, China; ^2^Laboratory of Theoretical Biophysics, School of Physical Science and Technology, Inner Mongolia University, Hohhot 010021, China; ^3^Gordon Life Science Institute, Boston, MA 02478, USA; ^4^Department of Physics, School of Sciences Center for Genomics and Computational Biology, Hebei United University, Tangshan 063000, China; ^5^Center of Excellence in Genomic Medicine Research (CEGMR), King Abdulaziz University, Jeddah 21589, Saudi Arabia

## Abstract

Conotoxins are small disulfide-rich neurotoxic peptides, which can bind to ion channels with very high specificity and modulate their activities. Over the last few decades, conotoxins have been the drug candidates for treating chronic pain, epilepsy, spasticity, and cardiovascular diseases. According to their functions and targets, conotoxins are generally categorized into three types: potassium-channel type, sodium-channel type, and calcium-channel types. With the avalanche of peptide sequences generated in the postgenomic age, it is urgent and challenging to develop an automated method for rapidly and accurately identifying the types of conotoxins based on their sequence information alone. To address this challenge, a new predictor, called iCTX-Type, was developed by incorporating the dipeptide occurrence frequencies of a conotoxin sequence into a 400-D (dimensional) general pseudoamino acid composition, followed by the feature optimization procedure to reduce the sample representation from 400-D to 50-D vector. The overall success rate achieved by iCTX-Type via a rigorous cross-validation was over 91%, outperforming its counterpart (RBF network). Besides, iCTX-Type is so far the only predictor in this area with its web-server available, and hence is particularly useful for most experimental scientists to get their desired results without the need to follow the complicated mathematics involved.

## 1. Introduction

**Figure 1:**
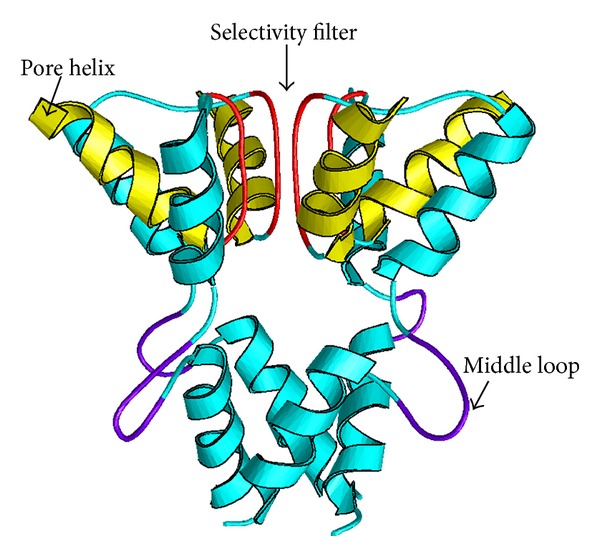
A ribbon drawing to show the human potassium (K) channel. Reproduced from Chou [6] with permission.

**Figure 2:**
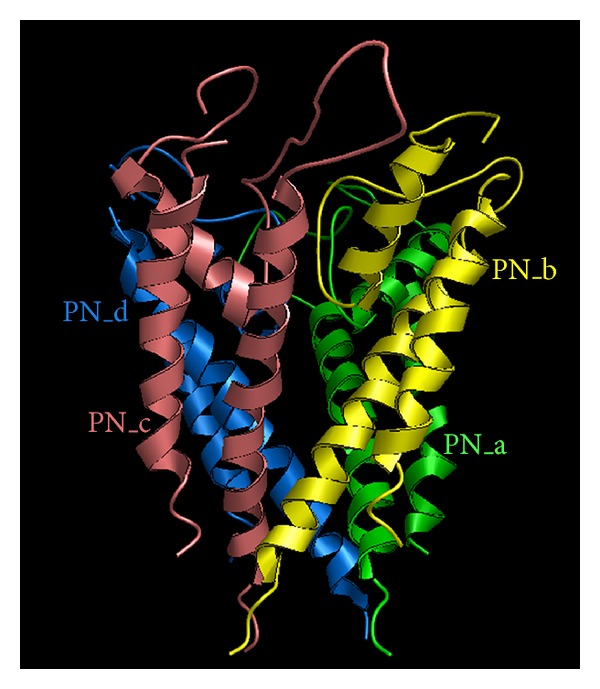
A ribbon drawing to show the human sodium (Na) channel. Reproduced from Chou [6] with permission.

**Figure 3:**
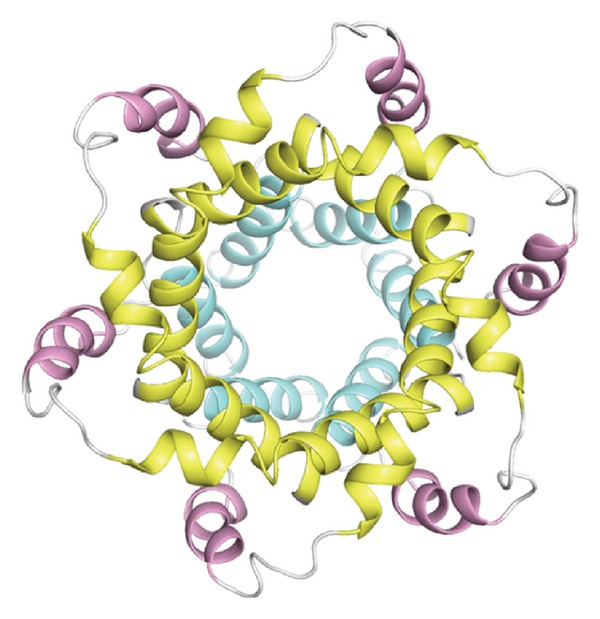
A ribbon drawing to show the calcium (Ca) channel from hepatitis C virus. Reproduced from [4] with permission.

Being peptides consisting of about 10 to 30 amino acid residues, conotoxins are toxins secreted by cone snails for capturing prey and securing themselves. This kind of toxins can bind to various targets, such as G protein-coupled receptors (GPCRs), nicotinic acetylcholine, and neurotensin receptors. In particular, they display extremely high specificity and affinity for ion channels. Ion channels represent a class of membrane spanning protein pores that mediate the flux of ions in a variety of cell types. There are over 300 types of ion channels in a living cell [1]. Many crucial functions in life, such as heartbeat, sensory transduction, and central nervous system response, are controlled by cell signaling via various ion channels. Ion channel dysfunction may lead to a number of diseases, such as epilepsy, arrhythmia, and type II diabetes. These kinds of diseases are primarily treated with the drugs that modulate the ion channels concerned. Ion channels are also the important targets for treating virus diseases (see, e.g., [2–4]). Owing to their importance to human being's life, ion channels have become the 2nd most frequent targets for drug development, just next to GPCRs (G protein-coupled receptors) [5]. The following three kinds of ion channels are usually the targets by conotoxins: potassium (K) channel (Figure 1), sodium (Na) channel (Figure 2), and calcium (Ca) channel (Figure 3). Based on their functions and targeting objects, conotoxins can be classified into the following three types: (i) K-channel-targeting type; (ii) Na-channel-targeting type; and (iii) Ca-channel-targeting type.

Although conotoxins are lethally venomous because of blocking the transmission of nerve impulses, they have been widely used to treat chronic pain, epilepsy, spasticity, and cardiovascular diseases. Therefore, conotoxins have been regarded as important pharmacological tools for neuroscience research.

It has been estimated that there are more than 100,000 kinds of conotoxins secreted by over 700 kinds of* Conus* in the world [8]. However, relatively much fewer conotoxins (about 3,000 peptides) have been experimentally confirmed and reported in literature and databases. Moreover, the records about the functions of conotoxins in public databases are no more than 300 items. Hence, developing a computational method to predict the functions of conotoxins has become a challenging task.

In a pioneer work, Mondal et al. [9] proposed a method for predicting conotoxin superfamilies by using the pseudoamino acid composition approach [10, 11]. Subsequently, a series of studies have been reported in predicting conotoxin superfamilies (see, for example, [12–15]). All these methods yielded quite encouraging results, and each of them did play a role in stimulating the development of this area. However, none of these methods can be used to predict the types of conotoxins defined according to their targeting ion-channels. For instance, both delta-conotoxin-like Ac6.1 (UniProt accession number: P0C8V5) [16] and omega-conotoxin-like Ai6.2 [17] (UniProt accession number: P0CB10) belong to the conotoxin O1 superfamily. However, the former targets the voltage-gated sodium channels, while the latter targets the voltage-gated calcium channels.

To deal with this problem, recently, a method was developed [7] to identify conotoxins among the aforementioned three types by using their sequence information alone. However, further work is needed in this regard due to the following reasons. (i) The prediction quality can be further improved. (ii) No web server for the prediction method in [7] was provided, and hence its usage is quite limited, especially for the majority of experimental scientists.

The present study was devoted to develop a new predictor for identifying the conotoxins' types from the above two aspects.

As elaborated in a comprehensive review [18] and conducted by a series of recent publications [19–28], to establish a really useful statistical predictor for a biological system, we need to consider the following procedures: (i) construct or select a valid benchmark dataset to train and test the predictor; (ii) formulate the biological samples with an effective mathematical expression that can truly reflect their intrinsic correlation with the target to be predicted; (iii) introduce or develop a powerful algorithm (or engine) to operate the prediction; (iv) properly perform cross-validation tests to objectively evaluate the anticipated accuracy of the predictor; (v) establish a user-friendly web server for the predictor that is accessible to the public. In what follows, let us describe how to deal with these procedures one by one.

## 2. Materials and Methods

### 2.1. Benchmark Dataset

The sequences of conotoxins and their functions were collected from the UniProt [29]. To ensure its quality, the benchmark dataset was constructed strictly according to the following criteria. (i) Included were only those peptides annotated with “conotoxin” and with the keyword of potassium, calcium, or sodium in their functional ontologies. (ii) Included were only those conotoxins with clear functional annotations based on experiment results. In other words, we excluded those annotated with “uncertain,” “predicted,” or “inferred from homology” because of lacking confidence. (iii) Excluded were those that were annotated with “immature” due to the incompleteness. (iv) Excluded were also those that contained any invalid amino acid codes, such as “B,” “X,” and “Z”. After going through the above procedures, we obtained 195 conotoxins, of which 37 belonged to the K-channel-targeting type, 86 to the Na-channel-targeting type, and 72 to the Ca-channel-targeting type.

As elaborated in a comprehensive review [18], a benchmark dataset containing many redundant samples with high similarity would lack statistical representativeness. A predictor, if trained and tested by a benchmark dataset with many homologous sequences, might yield misleading results with overestimated accuracy [30]. To remove the homologous sequences from the benchmark dataset, a cutoff threshold of 25% was recommended [31] to exclude those protein/peptide sequences from the benchmark datasets that had ≥25% pairwise sequence identity to any other sample in the same subset. However, in this study we did not use such a stringent criterion because the currently available data did not allow us to do so. Otherwise, the numbers of peptides for some subsets would be very few to have statistical significance. As a compromise, we set the cutoff threshold at 80% and used the CD-HIT software [32] to remove those conotoxin samples that had ≥80% sequence identity to any other in a same subset. After such a screening procedure, we obtained 112 conotoxin samples for the benchmark dataset *S*, as formulated as follows:

(1) $$\begin{array}{c} S^{}=S_{\text{K}}^{}\cup S_{\text{Na}}^{}\cup S_{\text{Ca}}^{}, \end{array}$$

where the subset *S*_K_ contains 24 conotoxin samples of K-channel-targeting type, *S*_Na_ contains 43 samples of Na-channel-targeting type, and *S*_Ca_ contains 45 samples of Ca-channel-targeting type, while the symbol ∪ represents the union in the set theory. The codes of 112 conotoxins and their sequences are given in Supporting Information S1 (see Supplementary Material available online at http://dx.doi.org/10.1155/2014/286419).

Likewise, we also constructed an independent dataset *S* ^Ind^ as formulated by

(2) $$\begin{array}{c} S_{}^{\text{Ind}}=S_{\text{K}}^{\text{Ind}}\cup S_{\text{Na}}^{\text{Ind}}\cup S_{\text{Ca}}^{\text{Ind}}, \end{array}$$

where *S*_K_^Ind^ contains 12 K-conotoxins, *S*_Na_^Ind^ contains 37 Na-conotoxins, and *S*_Ca_^Ind^ contains 21 Ca-conotoxins. None of the samples in the independent dataset occurs in the dataset *S* of (1), and their detailed sequences are given in Supporting Information S2.

For simplicity, hereafter, let us use “K-conotoxin,” “Na-conotoxin,” and “Ca-conotoxin” to represent K-channel-targeting type conotoxin, Na-channel-targeting type conotoxin, and Ca-channel-targeting type conotoxin, respectively.

### 2.2. The Dipeptide Mode of Pseudoamino Acid Composition

Given a conotoxin peptide** P **with L amino acids, how do we translate it into a mathematical expression for statistical prediction? This is one of the first important problems to develop a sequence-based predictor for identifying the type of a conotoxin. The most straightforward way to formulate the sample of a conotoxin peptide** P** with L residues is to use its entire amino acid sequence, as can be formulated by

(3) $$\begin{array}{c} P={\text{R}}_{1}{\text{R}}_{2}{\text{R}}_{3}{\text{R}}_{4}\cdots {\text{R}}_{\text{L}}, \end{array}$$

where R_1_ represents the 1st residue of the conotoxin peptide and R_2_ the 2nd residue of the peptide and so forth. Subsequently, we can utilize various sequence similarity search based tools, such as BLAST [33], to perform statistical prediction. Although this kind of sequence model was very straightforward and intuitive, unfortunately, it failed to work when a query conotoxin peptide did not have significant similarity to any of the peptide sequences in the training dataset. Thus, investigators turned to use vectors to represent the peptide samples. Another reason for them to do so is that the statistical samples in vector format are much easier to be handled than in sequence format by many existing operation engines, such as the correlation angle approach [34], covariance discriminant (CD) [27, 35–37], neural network [38–40], optimization approach [41], support vector machine (SVM) [22, 23, 42, 43], random forest [44, 45], conditional random field [20], nearest neighbor (NN) [46, 47]; K-nearest neighbor (KNN) [30], OET-KNN [48–50], fuzzy K-nearest neighbor [25, 51–55], ML-KNN algorithm [56], and SLLE algorithm [36].

The simplest vector used to represent a peptide or protein sample is its amino acid composition (AAC), as given as follows:

(4) $$\begin{array}{c} P={\left[\begin{array}{cccc} f_{1}^{} & f_{2}^{} & \cdots & f_{20}^{} \end{array}\right]}^{T}, \end{array}$$

where *f*_*i*_ (*i* = 1,2,…, 20) is the normalized occurrence frequency of the *i*th type of native amino acid in the peptide chain and **T** is the transpose operator. The AAC model was used by many in predicting various contributes of proteins (see, e.g., [41, 57–59]). However, as we can see from (4), when using AAC to represent a peptide or protein sample, all its sequence order information would be completely lost and hence limit the prediction quality.

How can we formulate a peptide or protein sequence with a vector yet still keep considerable sequence order information? As reported in many recent publications, in order to incorporate the sequence order information, the pseudoamino acid composition [10, 11] or Chou's PseAAC [60] was proposed. Since the concept of PseAAC was proposed in 2001 [10], it has been penetrating into almost all the fields of protein attribute predictions (see, e.g., [61–78]). Recently, the concept of PseAAC was further extended to represent the feature vectors of DNA and nucleotides [19, 21, 23, 27, 79], as well as other biological samples (see, e.g., [80–82]). Because it has been widely and increasingly used, in addition to the web server “PseAAC” [83] built in 2008, recently three types of powerful open access software, called “PseAAC-Builder” [84], “propy” [85], and “PseAAC-General” [86], were established: the former two are for generating various modes of Chou's special PseAAC, while the 3rd one is for those of Chou's general PseAAC.

According to a comprehensive review [18], the general PseAAC is formulated by

(5) $$\begin{array}{c} P={\left[\begin{array}{cccccc} \psi_{1} & \psi_{2} & \cdots & \psi_{u} & \cdots & \psi_{\Omega } \end{array}\right]}^{T}, \end{array}$$

where the component *ψ*_*u*_ (*u* = 1,2,…, *Ω*) and the dimension *Ω* will depend on how to extract the features from the peptide sequences concerned. For the current study, since the conotoxin sequences are not long (about 10–30 residues), we could just consider the sequence order information between two most contiguous amino acid residues. Thus, the dimension of the vector **P** in (5) is *Ω* = 20 × 20 = 400 and each of the components therein is given by

(6) $$\begin{array}{c} \psi_{u}^{}=\{\begin{array}{cc} f\left(\text{AA}\right) & \text{when}\;u=1\\ f\left(\mathrm{AC}\right) & \text{when}\;u=2\\ \;\;\vdots & \;\;\vdots \\ f\left(\text{AY}\right) & \text{when}\;u=20\\ f\left(\text{CA}\right) & \text{when}\;u=21\\ \;\;\vdots & \;\;\vdots \\ f\left(\text{YW}\right) & \text{when}\;u=399\\ f\left(\text{YY}\right) & \text{when}\;u=400, \end{array} \end{array}$$

where A, C,…, W, Y are, respectively, the single letter codes of 20 native amino acids, *f*(AA) is the occurrence frequency for the dipeptide AA in the conotoxin sequence (see (3)), and *f*(AC⁡) is for the dipeptide AC and so forth. The formulation defined by (5)-(6) is actually the dipeptide mode of PseAAC, which can be automatically generated by the PseAAC server [83] for a given peptide or protein sequence.

### 2.3. Feature Selection

The original raw features usually contain the redundant information and noise that may negatively affect the prediction quality [87]. Using the feature selection techniques to optimize the feature set can not only enhance the prediction accuracy but also provide useful insights for in-depth understanding of the action mechanism of conotoxins. According to the feature selection algorithm [87], the *F*-score function is defined by

(7) $$\begin{array}{c} F\left(i\right)=\frac{\sum_{k=1}^{3}{\left({\overset{-}{f}}_{i}^{\;k}-{\overset{-}{f}}_{i}\right)}^{2}}{\sum_{k=1}^{3}\left(1/\left(N_{k}-1\right)\right)\sum_{j=1}^{N_{k}}{\left(f_{ij}^{k}-{\overset{-}{f}}_{i}^{\;k}\right)}^{2}}, \end{array}$$

where ${\overset{-}{f}}_{i}^{\;k}$ is the average frequency of the *i*th feature in the *k*th dataset, ${\overset{-}{f}}_{i}$ the average frequency of the *i*th feature in the all datasets concerned, *f*_*ij*_^*k*^ is the frequencies of the *i*th feature of the *j*th sequence in the *k*th dataset, and *N*_*k*_ is the number of peptide samples in the *k*th dataset. The program called “fselect.py” was downloaded from http://www.csie.ntu.edu.tw/~cjlin/libsvmtools to calculate *F*-score defined in (7).

The larger the *F*-score is, the more likely it has a better discriminative capability [87]. Accordingly, we ranked the 400 dipeptides in (5) according to their *F*-scores. Subsequently, based on the ranked dipeptides, we performed the incremental feature selection (IFS) strategy to find an optimal subset of features that yielded the highest predictive accuracy. During the IFS procedure, the feature subset started with one feature with the highest *F*-score. A new feature subset was composed when one more feature with the second highest *F*-score was added. By adding these features sequentially from the higher to lower ranks, 400 feature sets would be obtained. The *τ*th feature set can be formulated as

(8) $$\begin{array}{c} S_{\tau }=\left\{f_{1},f_{2},\ldots ,f_{\tau }\right\},\;\left(1\leq \tau \leq 400\right). \end{array}$$

For each of the 400 feature sets, a prediction model based on the proposed predictive algorithm was constructed and examined with the jackknife cross-validation on the benchmark dataset. By doing so, we obtained an IFS curve in a 2D (dimensional) Cartesian coordinate system with index *τ* as the abscissa (or X-coordinate) and the overall accuracy as the ordinate (or Y-coordinate). The optimal feature set is expressed as

(9) $$\begin{array}{c} S_{\Theta }=\left\{f_{1},f_{2},\ldots ,f_{\Theta }\right\}. \end{array}$$

with which the IFS curve reached its peak. In other words, in the 2D coordinate system, when *X* = Θ, the value of the overall accuracy was the maximum. Thus, we used the Θ features to build the final predictor.

### 2.4. Support Vector Machine (SVM)

The classification algorithm used in this work was the support vector machine (SVM). The SVM has been widely used in the realm of bioinformatics (see, e.g., [19, 22, 23, 88–90]). Its basic principle is to transform the input vector into a high-dimension Hilbert space and seek a separating hyperplane with the maximal margin in this space by using the decision function:

(10) $$\begin{array}{c} f\left(\overset{\to }{X}\right)=\mathrm{sgn}\left(\overset{N}{\underset{i=1}{\sum }}y_{i}\alpha_{i}\cdot K\left(\overset{\to }{X},\overset{\to }{X_{i}}\right)+b\right), \end{array}$$

where $\overset{\to }{X_{i}}$ is the *i*th training vector, the *y*_*i*_ represents the type of the *i*th training vector, and $K(\overset{\to }{X},\overset{\to }{X_{i}})$ is a kernel function which defines an inner product in a high dimensional feature space. Because of its effectiveness and speed in nonlinear classification process, the radial basis kernel function (RBF) $K(\overset{\to }{X_{i}},\overset{\to }{X_{j}})=\exp(-\gamma {||\vec{X_{i}}-\vec{X_{j}}||}^{2}\;)\;$ was used in the current work. The original SVM was designed for two-class problems. For multiclass problems, several strategies such as one-versus-rest (OVR), one-versus-one (OVO), and DAGSVM have been applied to extend the traditional SVM. In the present study, we used the OVO strategy for multiclass prediction. The concrete SVM software (LibSVM) was downloaded from http://www.csie.ntu.edu.tw/~cjlin/libsvm. A grid search method was used to optimize the regularization parameter *C* and kernel parameter via the jackknife cross-validation. The search spaces for *C* and *γ* are [2^15^, 2^−5^] and [2^−5^, 2^−15^] with steps of 2^−1^ and 2, respectively. For more details about SVM, see a monograph [91].

## 3. Results and Discussion

### 3.1. Test Method and Criteria

In statistical prediction, the independent dataset test, subsampling or K-fold crossover test and jackknife test are the three cross-validation methods often used to check a predictor for its accuracy [92]. However, among the three test methods, the jackknife test is deemed the least arbitrary that can always yield a unique result for a given benchmark dataset [18]. Accordingly, the jackknife test has been increasingly used and widely recognized by investigators to examine the quality of various predictors (see, e.g., [19, 21, 73, 75, 93–95]). Therefore, in this study we also adopted the jackknife test.

In addition to an objective test method, we also need a set of metrics to reasonably measure the test outcome. Here, let us use the criterion proposed in [96, 97] to develop a set of more intuitive and easier-to-understand metrics; that is, the correct rates Λ^K^ in predicting K-conotoxins, Λ^Na^ in predicting Na-conotoxins, and Λ^Ca^ in predicting Ca-conotoxins are defined by

(11) ΛK=NK−NNaK−NCaKNK, for  the  K-conotoxinsΛNa=NNa−NKNa−NCaNaNK, for  the  Na-conotoxinsΛCa=NCa−NKCa−NNaCaNCa, for  the  Ca-conotoxins,

where *N* ^K^ is the total number of the K-conotoxins investigated, while *N*_Na_^K^ is the number of the K-conotoxins incorrectly predicted as the Na-conotoxins, and *N*_Ca_^K^ is the number of the K-conotoxins incorrectly predicted as the Ca-conotoxins; *N* ^Na^ is the total number of the Na-conotoxins investigated, while *N*_K_^Na^ is the number of the Na-conotoxins incorrectly predicted as the K-conotoxins and *N*_Ca_^Na^ is the number of the Na-conotoxins incorrectly predicted as the Ca-conotoxins; and *N* ^Ca^ is the total number of the Ca-conotoxins investigated, while *N*_Na_^Ca^ is the number of the Ca-conotoxins incorrectly predicted as the Na-conotoxins and *N*_K_^Ca^ is the number of the Ca-conotoxins incorrectly predicted as the K-conotoxins. From (11), it follows that

(12) OA=Λ=1−NNaK+NCaK+NKNa+NCaNa+NNaCa+NKCaNK+NNa+NCaAA=ΛK+ΛNa+ΛCa3,

where OA stands for the overall accuracy and AA for the average accuracy.

### 3.2. The Optimal Features

As mentioned above, it would be no good for a sample vector to contain either too few or too many features. This is because the former would limit the prediction quality due to lack of information, while the latter would generate a lot of noise due to redundancy. Therefore, we should find a set of optimal features, for which there is minimal redundancy among themselves but maximal relevancy to the target to be predicted. In the present study, such an optimal feature-set is none but (9).

**Figure 4:**
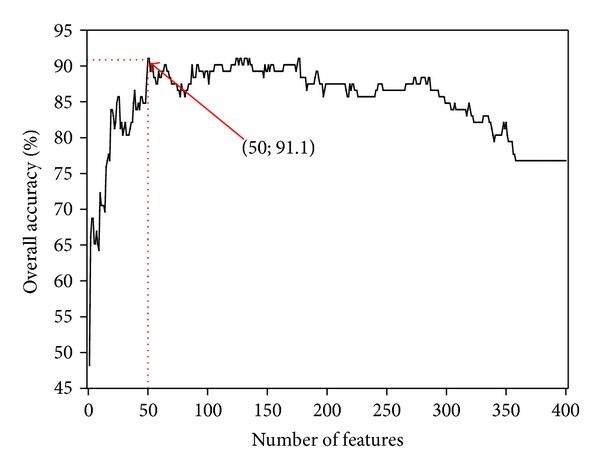
A plot to show the IFS curve, where the abscissa and ordinate axis denote the number of features and the overall accuracy, respectively. As shown in the figure, the value of the overall accuracy reached its peak (91.1%) when the top-ranked 50 dipeptide features were taken into account.

Shown in Figure 4 is the IFS curve for the value of OA against the number of the counted features, as described in Section 2.3. As can be seen from there, the value of OA reached its peak of 91.1% when the top-ranked 50 dipeptides (Table 1) were taken into account.

The predictor thus obtained via the aforementioned procedures is called “iCTX-Type,” where “i” stands for “identify” and “CTX” for “conotoxin.”

A comparison of the current predictor iCTX-Type with the one in [7] (i.e., to the best of our knowledge, it is the only existing predictor in this area) is given in Table 2, from which we can see the following. (i) For four of the five metrics defined in (10)-(11), iCTX-Type yielded higher scores than the method in [7]. Particularly, iCTX-Type achieved higher overall accuracy (OA) and average accuracy (AA). (ii) Compared with the method of [7] using 70 features, only 50 features were used in the present method (Table 1), indicating that the iCTX-Type is more efficient in excluding redundancy and noise as well as in capturing the core features.

To further verify the performance of the current predictor, iCTX-Type was also used to identify the samples in the independent dataset *S* ^Ind^ (see Supporting Information S2), and the success rates (see (11)) thus obtained were 91.7%, 91.9%, and 90.5% for K-, Na-, and Ca-conotoxins, respectively. These results are fully consistent with those obtained by the jackknife test as given in Table 2, furtherindicating that the new predictor iCTX-Type is quite promising and holds a high potential to become a useful tool for in-depth studying ion channel-targeted conotoxins.

To enhance the value of its practical applications [98], a web server for the new iCTX-Type predictor was established as described below.

### 3.3. Web-Server Guide

For the convenience of the vast majority of experimental scientists, below a step-by-step guide is provided for how to use the web server to get the desired results without the need to follow the mathematic equations that were presented in this paper just for the integrity in developing the predictor.

**Figure 5:**
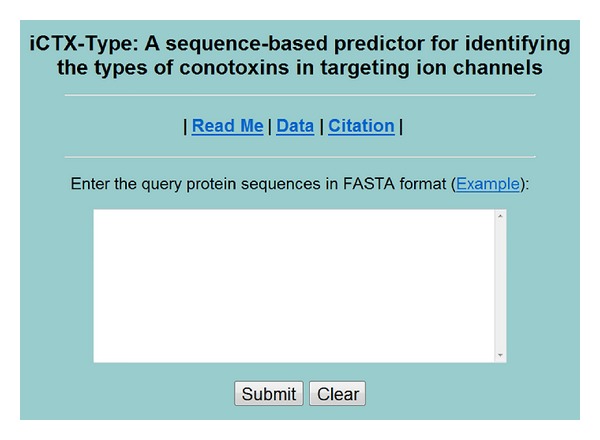
A screenshot to show the top page of the iCTX-Type web server. Its website address is http://lin.uestc.edu.cn/server/iCTX-Type.

*Step 1.* Open the web server at http://lin.uestc.edu.cn/server/iCTX-Type and you will see the top page of iCTX-Type on your computer screen, as shown in Figure 5. Click on the Read Me button to see a brief introduction about the predictor and the caveat when using it. 

*Step 2.* Either type or copy/paste the query peptide sequences into the input box at the center of Figure 5. The input sequence should be in the FASTA format. A sequence in FASTA format consists of a single initial line beginning with a greater-than symbol “>” in the first column, followed by lines of sequence data. The words right after the “>” symbol in the single initial line are optional and only used for the purpose of identification and description. All lines should be no longer than 120 characters and usually do not exceed 80 characters. The sequence ends if another line starting with a “>” appears; this indicates the start of another sample sequence. Example sequences in FASTA format can be seen by clicking on the Example button right above the input box.

*Step 3.* Click on the Submit button to see the predicted result. For instance, when using the three peptide sequences as an input and clicking the Submit button, you will see the following shown on the screen of your computer: the outcome for the 1st query example is “Ca-conotoxin”; the outcome for the 2nd query sample is “K-conotoxin”; the outcome for the 3rd query sample is “Na-conotoxin.” All these results are fully consistent with the experimental observations. It takes only a few seconds for the above computation before the predicted result appears on your computer screen; the more number of query sequences, the longer time it usually needs.

*Step 4.* Click on the Data button to download the benchmark datasets used to train and test the iCTX-Type predictor.

*Step 5.* Click on the Citation button to find the relevant papers that document the detailed development and algorithm of iCTX-Type.

*Caveats.* The input query sequences must be formed by the single-letter codes of the 20 native amino acids; any other characters such as “B,” “X,” “U,” and “Z” are invalid and should not be part of the peptide sequence.

## 4. Conclusion

It is anticipated that iCTX-Type may become a useful high throughput tool for both basic research and drug development, particularly for in-depth investigation into the mechanisms of ion-channels and developing new drugs to treat chronic pain, epilepsy, spasticity, and cardiovascular diseases, among others.

It is instructive to point out that since the binding of conotoxins to ion-channel is highly selective and specific, the information obtained by iCTX-Type in identifying the types of conotoxins may be also very useful for designing ion channel inhibitors according to the Chou's distorted key theory as elaborated in [99] and briefed in a Wikipedia article at http://en.wikipedia.org/wiki/Chou's_distorted_key_theory_for_peptide_drugs.

## Supplementary Material

Supporting Information S1: The benchmark dataset *𝕊* contains 112 conotoxins, of which 24 belong to K-channel-targeting type, 43 to Na-channel-targeting type, and 45 to Ca-channel-targeting type.
Supporting Information S2: The independent dataset *𝕊* ^Ind^ contains 70 conotoxins, of which 12 are of K-channel-targeting type, 37 of Na-channel-targeting type, and 21 of Ca-channel-targeting type. None of the samples listed here occurs in benchmark dataset *𝕊*.

## Figures and Tables

**Table 1:** List of the 50 optimal features or dipeptides derived according to (7)–(9) as elaborated in the Section 2.3.

AA	AS	CC	CH	CS	DH	DN	EN	GA	GH
GL	GT	GY	HA	HL	HS	IY	KD	KK	KM
KP	LN	LV	MC	MY	ND	NQ	NS	PI	QK
QT	RC	RD	RF	RN	RT	RW	SC	SG	TE
TF	TT	VV	WG	WI	YD	YH	YL	YT	YY

**Table 2:** Comparison of the current method with the one in [7] by the jackknife test on the same benchmark dataset (Supporting Information S1) according to the metrics defined in (11)-(12).

Method	Number of features counted	Λ^K^ (%)	Λ^Na^ (%)	Λ^Ca^ (%)	AA (%)	OA (%)
RBF network^a^	70	91.7	88.4	88.9	89.7	89.3
iCTX-Type^b^	50	83.3	97.8	89.8	90.3	91.1

^a^See [7].

^b^This paper.

## References

[1] Gabashvili IS, Sokolowski BH, Morton CC, Giersch AB (2007). Ion channel gene expression in the inner ear. *Journal of the Association for Research in Otolaryngology*.

[2] Schnell JR, Chou JJ (2008). Structure and mechanism of the M2 proton channel of influenza A virus. *Nature*.

[3] Pielak RM, Schnell JR, Chou JJ (2009). Mechanism of drug inhibition and drug resistance of influenza A M2 channel. *Proceedings of the National Academy of Sciences of the United States of America*.

[4] OuYang B, Xie S, Berardi MJ (2013). Unusual architecture of the p7 channel from hepatitis C virus. *Nature*.

[5] Xiao X, Min JL, Wang P (2013). Predict drug-protein interaction in cellular networking. *Current Topics in Medicinal Chemistry*.

[6] Chou K-C (2004). Insights from modeling three-dimensional structures of the human potassium and sodium channels. *Journal of Proteome Research*.

[7] Yuan LF, Ding C, Guo SH, Ding H, Chen W, Lin H (2013). Prediction of the types of ion channel-targeted conotoxins based on radial basis function network. *Toxicology in Vitro*.

[8] Daly NL, Craik DJ (2009). Structural studies of conotoxins. *IUBMB Life*.

[9] Mondal S, Bhavna R, Mohan Babu R, Ramakumar S (2006). Pseudo amino acid composition and multi-class support vector machines approach for conotoxin superfamily classification. *Journal of Theoretical Biology*.

[10] Chou K-C (2001). Prediction of protein cellular attributes using pseudo-amino acid composition. *Proteins*.

[11] Chou KC (2005). Using amphiphilic pseudo amino acid composition to predict enzyme subfamily classes. *Bioinformatics*.

[12] Lin H, Li QZ (2007). Predicting conotoxin superfamily and family by using pseudo amino acid composition and modified Mahalanobis discriminant. *Biochemical and Biophysical Research Communications*.

[13] Yin JB, Fan YX, Shen HB (2011). Conotoxin superfamily prediction using diffusion maps dimensionality reduction and subspace classifier. *Current Protein and Peptide Science*.

[14] Laht S, Koua D, Kaplinski L, Lisacek F, Stöcklin R, Remm M (2012). Identification and classification of conopeptides using profile hidden Markov Models. *Biochimica et Biophysica Acta*.

[15] Koua D, Laht S, Kaplinski L (2013). Position-specific scoring matrix and hidden Markov model complement each other for the prediction of conopeptide superfamilies. *Biochimica et Biophysica Acta*.

[16] Gowd KH, Dewan KK, Iengar P, Krishnan KS, Balaram P (2008). Probing peptide libraries from Conus achatinus using mass spectrometry and cDNA sequencing: identification of *δ* and *ω*-conotoxins. *Journal of Mass Spectrometry*.

[17] Hillyard DR, Mcintosh MJ, Jones RM O-superfamily conotoxin peptides.

[18] Chou K-C (2011). Some remarks on protein attribute prediction and pseudo amino acid composition. *Journal of Theoretical Biology*.

[19] Guo SH, Deng EZ, Xu LQ, Ding H, Lin H, Chen W (2014). iNuc-PseKNC: a sequence-based predictor for predicting nucleosome positioning in genomes with pseudo k-tuple nucleotide composition. *Bioinformatics*.

[20] Xu Y, Ding J, Wu LY (2013). iSNO-PseAAC: predict cysteine S-nitrosylation sites in proteins by incorporating position specific amino acid propensity into pseudo amino acid composition. *PLoS ONE*.

[21] Qiu WR, Xiao X (2014). iRSpot-TNCPseAAC: identify recombination spots with trinucleotide composition and pseudo amino acid components. *International Journal of Molecular Sciences*.

[22] Liu B, Zhang D, Xu R (2014). Combining evolutionary information extracted from frequency profiles with sequence-based kernels for protein remote homology detection. *Bioinformatics*.

[23] Chen W, Feng PM, Lin H, Chou KC (2013). iRSpot-PseDNC: identify recombination spots with pseudo dinucleotide composition. *Nucleic Acids Research*.

[24] Xiao JL, Min X, Chou K-C (2013). iEzy-Drug: a web server for identifying the interaction between enzymes and drugs in cellular networking. *BioMed Research International*.

[25] Xiao X, Min JL, Wang P (2013). iCDI-PseFpt: identify the channel-drug interaction in cellular networking with PseAAC and molecular fingerprints. *Journal of Theoretical Biology C*.

[26] Xu Y, Shao XJ, Wu LY, Deng NY, Chou KC (2013). iSNO-AAPair: incorporating amino acid pairwise coupling into PseAAC for predicting cysteine S-nitrosylation sites in proteins. *PeerJ*.

[27] Chen W, Lin H, Feng PM, Ding C, Zuo YC (2012). iNuc-PhysChem: a sequence-based predictor for identifying nucleosomes via physicochemical properties. *PLoS ONE*.

[28] Xu Y, Wen X, Shao XJ, Deng NY (2014). iHyd-PseAAC: predicting hydroxyproline and hydroxylysine in proteins by incorporating dipeptide position-specific propensity into pseudo amino acid composition. *International Journal of Molecular Sciences*.

[29] Consortium TU (2012). Reorganizing the protein space at the Universal Protein Resource (UniProt). *Nucleic Acids Research*.

[30] Chou KC, Shen HB (2006). Predicting eukaryotic protein subcellular location by fusing optimized evidence-theoretic K-nearest neighbor classifiers. *Journal of Proteome Research*.

[31] Chou KC, Shen HB (2007). Recent progress in protein subcellular location prediction. *Analytical Biochemistry*.

[32] Fu L, Niu B, Zhu Z, Wu S, Li W (2012). CD-HIT: accelerated for clustering the next-generation sequencing data. *Bioinformatics*.

[33] Wootton JC, Federhen S (1993). Statistics of local complexity in amino acid sequences and sequence databases. *Computers and Chemistry*.

[34] Chou JJ (1993). A formulation for correlating properties of peptides and its application to predicting human immunodeficiency virus protease-cleavable sites in proteins. *Biopolymers*.

[35] Chou KC (2005). Prediction of G-protein-coupled receptor classes. *Journal of Proteome Research*.

[36] Wang M, Yang J, Xu ZJ, Chou KC (2005). SLLE for predicting membrane protein types. *Journal of Theoretical Biology*.

[37] Xiao X, Wang P, Chou K-C (2008). Predicting protein structural classes with pseudo amino acid composition: an approach using geometric moments of cellular automaton image. *Journal of Theoretical Biology*.

[38] Feng KY, Cai YD, Chou KC (2005). Boosting classifier for predicting protein domain structural class. *Biochemical and Biophysical Research Communications*.

[39] Cai YD, Chou KC (1999). Artificial neural network model for predicting *α*-turn types. *Analytical Biochemistry*.

[40] Thompson TB, Zheng C, Chou K-C (1995). Neural network prediction of the HIV-1 protease cleavage sites. *Journal of Theoretical Biology*.

[41] Zhang CT, Chou KC (1992). An optimization approach to predicting protein structural class from amino acid composition. *Protein Science*.

[42] Feng PM, Chen W, Lin H (2013). iHSP-PseRAAAC: identifying the heat shock protein families using pseudo reduced amino acid alphabet composition. *Analytical Biochemistry*.

[43] Xiao X, Wang P, Chou KC (2012). iNR-physchem: a sequence-based predictor for identifying nuclear receptors and their subfamilies via physical-chemical property matrix. *PLoS ONE*.

[44] Lin WZ, Fang JA, Xiao X, Chou KC (2011). iDNA-prot: identification of DNA binding proteins using random forest with grey model. *PLoS ONE*.

[45] Kandaswamy KK, Chou K-C, Martinetz T (2011). AFP-Pred: a random forest approach for predicting antifreeze proteins from sequence-derived properties. *Journal of Theoretical Biology*.

[46] Cai YD, Chou KC (2004). Predicting subcellular localization of proteins in a hybridization space. *Bioinformatics*.

[47] Chou KC, Cai YD (2006). Prediction of protease types in a hybridization space. *Biochemical and Biophysical Research Communications*.

[48] Shen H, Chou KC (2005). Using optimized evidence-theoretic K-nearest neighbor classifier and pseudo-amino acid composition to predict membrane protein types. *Biochemical and Biophysical Research Communications*.

[49] Chou KC, Shen HB (2007). Euk-mPLoc: a fusion classifier for large-scale eukaryotic protein subcellular location prediction by incorporating multiple sites. *Journal of Proteome Research*.

[50] Shen HB, Chou KC (2009). A top-down approach to enhance the power of predicting human protein subcellular localization: Hum-mPLoc 2.0. *Analytical Biochemistry*.

[51] Zhang TL, Ding YS, Chou KC (2008). Prediction protein structural classes with pseudo-amino acid composition: approximate entropy and hydrophobicity pattern. *Journal of Theoretical Biology*.

[52] Xiao X, Wang P, Chou KC (2011). GPCR-2L: predicting G protein-coupled receptors and their types by hybridizing two different modes of pseudo amino acid compositions. *Molecular BioSystems*.

[53] Shen HB, Yang J, Chou KC (2006). Fuzzy KNN for predicting membrane protein types from pseudo-amino acid composition. *Journal of Theoretical Biology*.

[54] Xiao X, Min JL, Wang P (2013). iGPCR-Drug: a web server for predicting interaction between GPCRs and drugs in cellular networking. *PLoS ONE*.

[55] Xiao X, Wang P, Lin W-Z, Jia J-H, Chou K-C (2013). iAMP-2L: a two-level multi-label classifier for identifying antimicrobial peptides and their functional types. *Analytical Biochemistry*.

[56] Chou KC (2013). Some remarks on predicting multi-label attributes in molecular biosystems. *Molecular Biosystems*.

[57] Nakashima H, Nishikawa K, Ooi T (1986). The folding type of a protein is relevant to the amino acid composition. *Journal of Biochemistry*.

[58] Cedano J, Aloy P, Pérez-Pons JA, Querol E (1997). Relation between amino acid composition and cellular location of proteins. *Journal of Molecular Biology*.

[59] Zhou G-P (1998). An intriguing controversy over protein structural class prediction. *Protein Journal*.

[60] Lin S-X, Lapointe J (2013). Theoretical and experimental biology in one. *Journal of Biomedical Science and Engineering (JBiSE)*.

[61] Zhou X-B, Chen C, Li Z-C, Zou X-Y (2007). Using Chou’s amphiphilic pseudo-amino acid composition and support vector machine for prediction of enzyme subfamily classes. *Journal of Theoretical Biology*.

[62] Nanni L, Lumini A (2008). Genetic programming for creating Chou’s pseudo amino acid based features for submitochondria localization. *Amino Acids*.

[63] Georgiou DN, Karakasidis TE, Nieto JJ, Torres A (2009). Use of fuzzy clustering technique and matrices to classify amino acids and its impact to Chou’s pseudo amino acid composition. *Journal of Theoretical Biology*.

[64] Esmaeili M, Mohabatkar H, Mohsenzadeh S (2010). Using the concept of Chou’s pseudo amino acid composition for risk type prediction of human papillomaviruses. *Journal of Theoretical Biology*.

[65] Mohabatkar H (2010). Prediction of cyclin proteins using chou’s pseudo amino acid composition. *Protein and Peptide Letters*.

[66] Sahu SS, Panda G (2010). A novel feature representation method based on Chou’s pseudo amino acid composition for protein structural class prediction. *Computational Biology and Chemistry*.

[67] Mohabatkar H, Mohammad Beigi M, Esmaeili A (2011). Prediction of GABA(A) receptor proteins using the concept of Chou’s pseudo-amino acid composition and support vector machine. *Journal of Theoretical Biology*.

[68] Mohammad Beigi M, Behjati M, Mohabatkar H (2011). Prediction of metalloproteinase family based on the concept of Chou’s pseudo amino acid composition using a machine learning approach. *Journal of Structural and Functional Genomics*.

[69] Mei S (2012). Multi-kernel transfer learning based on Chou’s PseAAC formulation for protein submitochondria localization. *Journal of Theoretical Biology*.

[70] Nanni L, Brahnam S, Lumini A (2012). Wavelet images and Chou’s pseudo amino acid composition for protein classification. *Amino Acids*.

[71] Nanni L, Lumini A, Gupta D, Garg A (2012). Identifying bacterial virulent proteins by fusing a set of classifiers based on variants of Chou’s Pseudo amino acid composition and on evolutionary information. *IEEE/ACM Transactions on Computational Biology and Bioinformatics*.

[72] Gupta MK, Niyogi R, Misra M (2013). An alignment-free method to find similarity among protein sequences via the general form of Chou’s pseudo amino acid composition. *SAR and QSAR in Environmental Research*.

[73] Hajisharifi Z, Piryaiee M, Mohammad Beigi M, Behbahani M, Mohabatkar H (2014). Predicting anticancer peptides with Chou’s pseudo amino acid composition and investigating their mutagenicity via Ames test. *Journal of Theoretical Biology*.

[74] Huang C, Yuan J (2013). Using radial basis function on the general form of Chou’s pseudo amino acid composition and PSSM to predict subcellular locations of proteins with both single and multiple sites. *Biosystems*.

[75] Huang C, Yuan JQ (2013). Predicting protein subchloroplast locations with both single and multiple sites via three different modes of Chou’s pseudo amino acid compositions. *Journal of Theoretical Biology*.

[76] Mohabatkar H, Mohammad Beigi M, Abdolahi K, Mohsenzadeh S (2013). Prediction of allergenic proteins by means of the concept of Chou’s pseudo amino acid composition and a machine learning approach. *Medicinal Chemistry*.

[77] Sarangi AN, Lohani M, Aggarwal R (2013). Prediction of essential proteins in prokaryotes by incorporating various physico-chemical features into the general form of Chou’s pseudo amino acid composition. *Protein and Peptide Letters*.

[78] Wan S, Mak MW, Kung SY (2013). GOASVM: a subcellular location predictor by incorporating term-frequency gene ontology into the general form of Chou’s pseudo-amino acid composition. *Journal of Theoretical Biology*.

[79] Chen W, Lei TY, Jin DC, Lin H (2014). PseKNC: a flexible web-server for generating pseudo K-tuple nucleotide composition. *Analytical Biochemistry*.

[80] Li B-Q, Huang T, Liu L, Cai Y-D, Chou K-C (2012). Identification of colorectal cancer related genes with mrmr and shortest path in protein-protein interaction network. *PLoS ONE*.

[81] Huang T, Wang J, Cai Y-D, Yu H, Chou K-C (2012). Hepatitis C virus network based classification of hepatocellular cirrhosis and carcinoma. *PLoS ONE*.

[82] Jiang Y, Huang T, Chen L, Gao YF, Cai Y, Chou K-C (2013). Signal propagation in protein interaction network during colorectal cancer progression. *BioMed Research International*.

[83] Shen H-B, Chou K-C (2008). PseAAC: a flexible web server for generating various kinds of protein pseudo amino acid composition. *Analytical Biochemistry*.

[84] Du P, Wang X, Xu C, Gao Y (2012). PseAAC-Builder: a cross-platform stand-alone program for generating various special Chou’s pseudo-amino acid compositions. *Analytical Biochemistry*.

[85] Cao DS, Xu QS, Liang YZ (2013). propy: a tool to generate various modes of Chou’s PseAAC. *Bioinformatics*.

[86] Du P, Gu S, Jiao Y (2014). PseAAC-General: fast building various modes of general form of Chou’s pseudo-amino acid composition for large-scale protein datasets. *International Journal of Molecular Sciences*.

[87] Chen YW LC, Guyon I, Nikravesh N, Gunn S, Zadeh L (2006). Combining SVMs with various feature selection strategies. *Feature Extraction*.

[88] Lin H, Ding H, Guo F-B, Huang J (2010). Prediction of subcellular location of mycobacterial protein using feature selection techniques. *Molecular Diversity*.

[89] Chou K-C, Cai Y-D (2002). Using functional domain composition and support vector machines for prediction of protein subcellular location. *The Journal of Biological Chemistry*.

[90] Cai Y-D, Zhou G-P, Chou K-C (2003). Support vector machines for predicting membrane protein types by using functional domain composition. *Biophysical Journal*.

[91] Cristianini N, Shawe-Taylor J (2000). *An Introduction of Support Vector Machines and Other Kernel-Based Learning Methodds*.

[92] Chou KC, Zhang CT (1995). Review: prediction of protein structural classes. *Critical Reviews in Biochemistry and Molecular Biology*.

[93] Zhou GP, Assa-Munt N (2001). Some insights into protein structural class prediction. *Proteins: Structure, Function and Genetics*.

[94] Chou K-C, Wu Z-C, Xiao X (2012). ILoc-Hum: using the accumulation-label scale to predict subcellular locations of human proteins with both single and multiple sites. *Molecular BioSystems*.

[95] Chou K-C, Wu Z-C, Xiao X (2011). iLoc-Euk: a multi-label classifier for predicting the subcellular localization of singleplex and multiplex eukaryotic proteins. *PLoS ONE*.

[96] Chou K-C (2001). Using subsite coupling to predict signal peptides. *Protein Engineering*.

[97] Chou KC (2001). Prediction of signal peptides using scaled window. *Peptides*.

[98] Chou KC, Shen HB (2009). Review: recent advances in developing web-servers for predicting protein attributes. *Natural Science*.

[99] Chou KC (1996). Review: prediction of human immunodeficiency virus protease cleavage sites in proteins. *Analytical Biochemistry*.

