# Supplementary material for: iCTX-Type: A Sequence-Based Predictor for Identifying the Types of Conotoxins in Targeting Ion Channels
Source: Biomed Res Int. 2014 Jun 1;2014:286419. doi: 10.1155/2014/286419 (PMC4058692; doi:10.1155/2014/286419)
Supplement: Supplementary file 1 — Supporting Information S1: The benchmark dataset 𝕊 contains 112 conotoxins, of which 24 belong to K-channel-targeting type, 43 to Na-channel-targeting type, and 45 to Ca-channel-targeting type. Supporting Information S2: The independent dataset 𝕊 Ind contains 70 conotoxins, of which 12 are of K-channel-targeting type, 37 of Na-channel-targeting type, and 21 of Ca-channel-targeting type. None of the samples listed here occurs in benchmark dataset 𝕊. [file 286419.f1.pdf]

Supporting Information S1. The benchmark dataset $\mathbb{S}$ contains 112 conotoxins, of which 24 belong to K-channel-targeting type, 43 to Na-channel-targeting type, and 45 to Ca-channel-targeting type.

1. The 24 conotoxins of K-channel-targeting type

>sp|P0CY82|13-48
APELVVTATTTCCGYDPMTICPPCMCTHSCPPKRKP
>sp|A3DT44|12-40
APWMVVTATTNCCGYTGPACHPCLCTQSC
>sp|P0CAQ2|12-47
QKELVVTATTTCCGYNPMTSCPRCMCDSSCNKKKKP
>sp|P0CAQ3|12-48
QKELVPSKITTCCGYSPGTACPSCMCTNTCKKKNKKP
>sp|P0CE75|39-75
QTWLVPSTITTCCGYDPGTMCPTCMCDNTCKPKPKKS
>sp|P0C829|39-80
QKELVPSVITTCCGYDPGTMCPPCRCTNSCPTKPKKPGRRND
>sp|P0C2C6|1-24
DCCGVKLEMCHPCLCDNSCKKSGK
>sp|P69500|27-59
SRCFPPGIYCTPYLPCCWGICCDTCRNVCHLRI
>sp|P0C258|27-57
CRAEGTYCENDSQCCLNECCWGGCGHPCRHP
>sp|P0C615|1-32
CRTEGMSCEENQQCCWRSCCRGECEAPCRFGP
>sp|C7DQY0|27-70
SRCFPPGIYCTPYLPCCWGICCGTCRNDNSSLTFLQFCLPFFFF
>sp|Q0N4U4|40-64
SPGSTICKMACRTGNGHKYPFCNCR
>sp|Q0N4U7|40-64
GPGSAICNMACRLGQGHMYPFCNCN
>sp|Q0N4U3|40-64
SSGSTVCKMMCRLGYGHLYPSCGCR
>sp|Q0N4U8|40-64
FPRPRICNLACRAGIGHKYPFCHCR
>sp|P0C6S2|1-27
GGVGRCIYNCMNSGGGLNFIQCKTMCY
>sp|P0C6S3|1-28
RWDVDQCIYYCLNGVVGYSYTECQTMCT
>sp|P0CG45|1-25
LPPCCTPPKKHCPAPACKYKPCCKS
>sp|P69769|51-74
LPSCCSLNLRLCPVPACKRNPCCT
>sp|P84713|1-13
FHGGSWYRFPWGY
>sp|P56633|46-72
CRIPNQKCFQHLDDCCSRKCNRFNKCV
>sp|P0CY85|24-87
SKRWTRPSVCNLPAESGTGTQSLKRFYYNSDKMQCRTFIYKGNGGNDNNFPRTYDCQ
KKCLYRP

>sp|P0C1X2|27-86
KDRPSLCDLPADSGSGTKAEKRIYYNSARKQCLRFDYTGQGGNENNFRRTYDCQRTC
LYT
>sp|P83047|1-9
GDCPWKPWC

2. The 43 conotoxins of Na-channel-targeting type

>sp|P58923|1-30
SCSGRDSRCPPVCCMGLMCSRGKCVSIYGE
>sp|P15472|1-34
ACSGRGSRCPPQCCMGLRCGRGNPQKCIGAHEDV
>sp|P0C256|1-43
GHVSCGKDGRACDYHADCCNCCLGGICKPSTSWIGCSTNVFLT
>sp|P0C257|1-45
GAVPCGKDGRQCRNHADCCNCCPIGTCAPSTNWILPGCSTGQFMT
>sp|P0C259|1-42
GCKKDRKPCSYQADCCNCCPIGTCAPSTNWILPGCSTGPFMA
>sp|Q7Z090|36-75
GPRCWVGRVHCTYHKDCCPSVCCFKGRCKPQSWGCWSGPT
>sp|P0C612|1-38
NWSWCSGSGEGCDYHSECCGERCCIESMCIGDGVACWP
>sp|Q7Z095|1-46
GPSFCKADEKPCEYHSDCCNCCLSGICAPSTNWILPGCSTSSFFKI
>sp|Q7Z096|37-79
GPSFCKADEKPCKYHADCCNCCLGGICKPSTSWIGCSTNVFLT
>sp|Q7Z0A5|1-44
GHVPCGKDGRKCGYHADCCNCCLSGICKPSTSWTGCSTSTVQLT
>sp|P0C349|1-22
RHGCCKGPKGCSSRECRPQHCC
>sp|C1J5M5|52-74
VTDRCCKGKRECGRWCRDHSRCC
>sp|P0C1T9|1-22
GRCCDVPNACSGRWCRDHAQCC
>sp|P0C1U1|1-22
GRCCEGPNGCSSRWCKDHARCC
>sp|P01523|51-72
RDCCTPPKKCKDRQCKPQRCCA
>sp|P0C195|1-16
CCNCSSKWCRDHSRCC
>sp|P58925|50-71
QRLCCGFPKSCRSRQCKPHRCC
>sp|P60207|7-28
QRCCNGRRGCSSRWCRDHSRCC
>sp|Q86DU6|52-71
QNCCNGGCSSKWCRDHARCC
>sp|C1J5M6|52-75
VGERCCKNGKRGCGRWCRDHSRCC
>sp|P0C1U0|1-25
QGCCGEPNLCFTRWCRNNARCCRQQ
>sp|P01524|1-22

RDCCTPPRKCKDRRCKPMKCCA
>sp|P0CH16|1-22
ERVCCGYPMSCKSRACKPSYCC
>sp|P0C8V3|1-23
QKCCTGKKGSCSGRACKNLRCCA
>sp|C1J5M7|52-77
IVDRCCNKGNGKRGCSRWCRDHSRCC
>sp|Q9BP55|25-41
CCKYGWTCVLGCSPCGC
>sp|P0C8V5|52-83
DECFSPGTFCGIKPGLCCSAWCYSFFCLTLTF
>sp|P0CB09|52-78
WCKQSGEMCNLLDQNCCEGYCIVLVCT
>sp|P0C8V6|52-83
DECYPPGTFCGIKPGLCCSERCFPFVCLSLEF
>sp|P58913|52-80
EACYAPGTFCGIKPGLCCSEFCLPGVCFG
>sp|Q26443|52-82
ACSKKWEYCIVPILGFVYCCPGLICGPFVCV
>sp|P60179|1-26
CKQAGESCDIFSQNCCVGTCAFICIE
>sp|P69748|52-78
DGCSNAGAFCGIHPGLCCSEICIVWCT
>sp|P69749|52-78
DECSAPGAFCLIRPGLCCSEFCFFACF
>sp|P69750|52-82
YECYSTGTFCGINGGLCCSNLCLFFVCLTFS
>sp|P60513|1-32
DDCIKPYGFCSLPILKNGLCCSGACVGVCADL
>sp|Q9TWM7|49-77
VKPCRKEGQLCDPIFQNCCRGWNCVLFCV
>sp|P69753|52-83
DGCYNAGTFCGIRPGLCCSEFCFLWCITFVDS
>sp|P69755|52-83
DECYPPGTFCGIKPGLCCSAICLSFVCISFDF
>sp|P0CC15|1-32
DECFSPGTFCGFKPGLCCSARCFSLFCISLEF
>sp|A6YR20|43-87
DVCDSLVGGHCIHNGCWCDQEAPHGNCCDTDGCTAAWWCPGTKWD
>sp|Q1A3R1|51-62
DCCPAKLLCCNP
>sp|Q9U657|53-83
TCQRRWDFCPGALVGVITCCGGLICLGVMCI

3. The 45 conotoxins of Ca-channel-targeting type

>sp|Q1L777|22-37
GCCSHPACSVNHPELC
>sp|Q9U648|53-76
CYDGGTSCDSGIQCCSGWCIFVCL
>sp|Q5K0D6|43-78

ATDCIEAGNYCGPTVMKICCGFCSPYSKICMNYPKN
>sp|Q5K0D5|49-72
CRPSGSPCGVTSICCGRCSRGKCT
>sp|Q9XYZ1|47-74
TCNTPTQYCTLHRHCCSLYCHKTIHACA
>sp|Q5K0B9|52-80
VCIADDMPCGFGLFGGPLCCSGWCLFVCL
>sp|Q5K0C0|52-81
GCLPDEYFCGFSMIGALLCCSGWCLGICMT
>sp|Q9XZK2|46-70
CKAAGKPCSRIAYNCCTGSCRSGKC
>sp|Q9U651|52-76
CLDAGEICDFFFPTCCGYCILLFCA
>sp|Q9XZL1|53-81
YDCEPPGNFCGMIKIGPPCCSGWCFFACA
>sp|Q9U654|51-76
CVPYEGPCNWLTQNCCDATCVVFWCL
>sp|Q9XZK4|43-77
STSCMEAGSYCGSTTRICCGYCAYFGKKCIDYPSN
>sp|P0CB10|52-77
CTQSGELCDVIDPDCCNNFCIIFFCI
>sp|P0C8V8|46-70
CKGKGASCSRTMYNCCTGSCNRGKC
>sp|P0C831|46-71
CKGKGAPCRKTMYDCCSGSCGRRGKC
>sp|Q3YED6|53-82
DDECEPPGDFCGFFKIGPPCCSGWCFLWCA
>sp|P0CI41|1-13
NCPAGCRSQGCCM
>sp|P58917|46-70
CKSTGASCRRTSYDCCTGSCRSGRC
>sp|P01522|46-73
CKSPGSSCSPTSYNCCRSCNPYTKRCYG
>sp|Q9XYZ0|51-80
DCRPVGQYCGIPYEHNWRCCSQLCAIICVS
>sp|P28881|46-71
CKLKGQSCRKTSYDCCSGSCGRSGKC
>sp|P58920|46-72
CKSKGAKCSKLMYDCCSGSCSGTVGRC
>sp|Q9XZL3|51-82
NYCQEKWDYCPVPFLGSRYCCDGLFCTLFFCA
>sp|P05484|46-70
CKGKGAKCSRLMYDCCTGSCRSGKC
>sp|Q5K0C4|52-80
CDEEGTGCSSDSECCSGRCTPEGLFEFCE
>sp|P0C832|5-37
CMEAGSYCGSTTRICCGYCAYSASKNVCDYPSN
>sp|P56712|46-76
GCLEVDYFCGIPFANNGLCCSGNCVFVCTPQ
>sp|P58914|1-27
CKPPGSPCRVSSYNCCSSCKSYNKKCG

>sp|P58918|1-25
CKGKGASCRKTMYDCCRGSCRSGRC
>sp|P58919|1-26
CKGKGQSCSKLMYDCCTGSCSRRGKC
>sp|P0CY69|28-62
STSCVEAGSYCRPNVKLCCGFCSPYSKICMNFPKN
>sp|P0CY60|46-70
CKGPGAKCLKTMYDCCKYSCSRGRC
>sp|P05483|1-29
CKSPGTPCSRGMRDCCTSCLLYSNKCRRY
>sp|P58916|1-27
CKGKGAPCTRLMYDCCHGSCSSSKGRC
>sp|Q26350|4-28
CQGRGASCRKTMYNCCSGSCNRGRC
>sp|P56714|52-77
CKQADEPCDVFSLDCCTGICLGVCMW
>sp|P83301|1-33
EDCIAVGQLCVFWNIGRPCCSGLCVFACTVKLP
>sp|P0C248|1-8
GCPWDPWC
>sp|P0CY84|55-62
KCPWSPWC
>sp|P0C249|1-8
GCVLYPWC
>sp|Q2I2P3|55-62
GCPWEPWC
>sp|P62903|52-62
NESECPWHPWC
>sp|Q9BPG6|51-63
ECCEDGWCCTAAP
>sp|Q9BH84|51-78
VCVDGGTFCGFPKIGGPCCSGWCIFVCL
>sp|Q9BP99|52-84
DCRALGEYCGLPYVHNSRCCSQLCGFICVPESP
