# Supplementary material for: iCTX-Type: A Sequence-Based Predictor for Identifying the Types of Conotoxins in Targeting Ion Channels
Source: Biomed Res Int. 2014 Jun 1;2014:286419. doi: 10.1155/2014/286419 (PMC4058692; doi:10.1155/2014/286419)
Supplement: Supplementary file 2 [file 286419.f2.pdf]

Supporting Information S2. The independent dataset $\mathbb{S}^{\text{Ind}}$ contains 70 conotoxins, of which 12 are of K-channel-targeting type, 37 of Na-channel-targeting type, and 21 of Ca-channel-targeting type. None of the samples listed here occurs in the benchmark dataset $\mathbb{S}$.

1. The 12 conotoxins of K-channel-targeting type

>sp|P0C830|1-34
QKELVPSKTTTCCGYSPGTMCPSCMCTNTCPPQK
>sp|P0C1X1|39-74
APELVVTATTNCCGYNPMTICPPCMCTYSCPPKRKP
>sp|P0C828|39-68
QKSLVPSVITTCCGYDPGTMCPPCRCTNSC
>sp|P0CE76|39-75
APWLVPSTITTCCGYDPGSMCPPCMCNNTCKPKPKKS
>sp|P69501|27-59
SRCFPPGIYCTPYLPCCWGICCDTCRNVCHLRF
>sp|C7DQB7|27-59
SRCFPPGIYCTPYLPCCWGICCGTCRNVCHLRF
>sp|P0C252|1-31
CFPPGIYCTPYLPCCWGICCGTCRNVCHLRI
>sp|Q9U3Z3|27-57
CRAEGTYCENDSQCCLNECCWGGCGHPCRHP
>sp|Q7YZS9|27-59
SRCFPPGIYCTPYLPCCWGICCGTCRNVCHLRI
>sp|Q0N4U5|40-64
GPGSAICNMACRLEHGHLYPFCNCD
>sp|Q0N4U6|40-64
GPGSAICNMACRLEHGHLYPFCHCR
>sp|P84704|1-27
GGLGRCIYNCMNSGGGLSFIQCKTMCY

2. The 37 conotoxins of Na-channel-targeting type

>sp|P58928|1-30
APWLVPSQITTCCGYNPGTMCPSCMCTNTC
>sp|Q7Z0A6|1-42
GHVPCGKDGRKCGYHADCCNCCLSGICKPSTSWTGCSTSTFD
>sp|Q7Z099|1-45
GAVPCGKDGRQCRNHADCCNCCPIGTCAPSTNWILPGCSTGPFMT
>sp|P0C614|1-42
GHVPCGKDGRKCGYHADCCNCCLSGICKPSTSWTGCSTSTFD
>sp|Q7Z098|1-45
GAVPCGKDGRQCRNHADCCNCCPIGTCAPSTNWILPGCSTGQFMT
>sp|P0C610|1-43
GCKKDRKPCSYHADCCNCCLSGICAPSTNWILPGCSTSSFFKI
>sp|P0C611|1-46
GPSSCKADEEPCEYHADCCNCCLSGICAPSTNWILPGCSTSSFFKI
>sp|Q7Z094|1-46
GPSFCKADEKPCEYHADCCNCCLSGICAPSTNWILPGCSTSSFFKI
>sp|Q7Z092|1-46
GPSFCKANGKPCSYHADCCNCCLSGICKPSTNVILPGCSTSSFFRI

>sp|Q7Z091|1-41
GCKKDRKPCSYHADCCNCCLSGICAPSTNWILPGCSTSTFT
>sp|Q7Z0A2|1-42
GHVPCGKDGRKCGYHADCCNCCLSGICKPSTSWTGCSTSTFN
>sp|Q7Z0A1|1-42
GHVPCGKDGRKCGYHTHCCNCCLSGICKPSTSLIGCSTSSFT
>sp|Q7Z093|1-46
GPSFCKANGKPCSYHADCCNCCLSGICAPSTNWILPGCSTSSFFKI
>sp|Q7Z097|1-45
GAVPCGKDGRQCRNHADCCNCCPFGTCAPSTNRILPGCSTGMFLT
>sp|Q7Z0A0|1-43
GHVSCGKDGRACDYHADCCNCCLGGICKPSTSWIGCSTNVFLT
>sp|Q7Z0A4|1-44
GHVPCGKDRRKCGYHADCCNCCLSGICKPSTSWTGCSTSTFLLT
>sp|Q7Z0A3|1-44
GHVPCGKDGRKCGYHADCCNCCLSGICKPSTSWTGCSTSTFLLT
>sp|P0C1U2|1-22
QGCCNVPNGCSGRWCRDHAQCC
>sp|P0C8V2|1-22
RCCTGKKGSCSGRACKNLKCCA
>sp|P0C350|1-22
RHGCCKGPKGCSSRECRPQHCC
>sp|P0CE77|1-20
QNCCNGGCSSKWCKGHARCC
>sp|P05482|1-22
RDCCTPPKKCKDRRCKPLKCCA
>sp|P58926|1-17
CCKYGWTCLLGCSPCGC
>sp|P0C8V7|52-81
YECYSTGTFCGVNGGLCCSNLCLFFVCLFS
>sp|B2KJ30|54-78
CSNAGGFCGIHPGLCCSEICLVWCT
>sp|P18511|52-78
WCKQSGEMCNLLDQNCCDGYCIVLVCT
>sp|Q9U655|52-78
WCKQSGEMCNLLDQNCCDGYCIVLVCT
>sp|Q9XZK5|52-82
DGCSSGGTFCGIHPGLCCSEFCFLWCITFID
>sp|P56710|1-31
SKCFSPGTFCGIKPGLCCSVRCFSLFCISFE
>sp|P69757|52-82
DGCSSGGTFCGIRPGLCCSEFCFLWCITFID
>sp|P0CC13|1-32
DECFSPGTFCGTKPGLCCSARCFSFFCISLEF
>sp|P69752|1-29
EACYPPGTFCGIKPGLCCSELCLPAVCVG
>sp|P69754|52-83
EACYNAGSFCGIHPGLCCSEFCILWCITFVDS
>sp|P0CC14|1-32
DECFSPGTFCGIKPGLCCSARCLSFFCISLEF
>sp|P69756|1-32

EACYNAGTFCGIKPGLCCSAICLSFVCISFDF
>sp|P69751|52-78
YGCSNAGAFCGIHPGLCCSELCLVWCT
>sp|I1SB07|1-22
QGCCNGPKGCSSKWCRDHARCC

3. The 21 conotoxins of Ca-channel-targeting type

>sp|Q5K0D8|43-78
AADCIEAGNYCGPTVMKLCCGFCSPYSKICMNYPKN
>sp|Q9XZL0|53-76
CYDGGTSCDSGIQCCSGWCIFVCF
>sp|Q9U650|51-75
CLDAGEICDFFFPTCCGYCILLFCA
>sp|Q9XZK3|43-78
ATDCIEAGNYCGPTVMKICCGFCSPYSKICMNYPKN
>sp|Q9XZL2|51-76
CVPYEGPCNWLTQNCCDATCVVFWCL
>sp|Q9XZL4|49-72
CRPSGSPCGVTSICCGRCYRGKCT
>sp|Q3YEF3|52-76
CLDAGEVCDIFFPTCCGYCILLFCA
>sp|Q9XZL5|49-72
CRPSGSPCGVTSICCGRCSRGKCT
>sp|P0CB11|53-76
CYDGGTSCNTGNQCCSGWCIFLCL
>sp|P0C8V9|43-78
ATDCIEAGNYCGPTVMKICCGFCSPFSKICMNYPQN
>sp|P28880|49-72
CRSSGSPCGVTSICCGRCYRGKCT
>sp|Q5K0D3|51-77
SYCGSTTRICCGYCAYFGKKCIDYPSN
>sp|Q9XZK8|52-76
CLDAGEVCDIFFPTCCGYCILLFCA
>sp|P58915|1-26
CLSPGSSCSPTSYNCCRSCNPYSRKC
>sp|P56713|53-82
DDDCEPPGNFCGMIKIGPPCCSGWCFFACA
>sp|P05485|1-25
CKGKGASCHRTSYDCCTGSCNRGKC
>sp|P37300|3-28
CKGKGAPCRKTMYDCCSGSCGRRGKC
>sp|Q9U644|52-82
DCQEKWDFCPAPFFGSRYCCFGLFCTLFFCA
>sp|Q9U645|53-81
DDCEPPGNFCGMIKIGPPCCSGWCFFACA
>sp|Q9BPA5|51-78
DCVAGGHFCGFPKIGGPCCSGWCFFVCA
>sp|P0C250|1-8
GCPWDPWC
